# Standardizing Visual Control Devices for Tsetse Flies: East African Species *Glossina swynnertoni*

**DOI:** 10.1371/journal.pntd.0002063

**Published:** 2013-02-28

**Authors:** Furaha Mramba, Francis Oloo, Mechtilda Byamungu, Thomas Kröber, Andrew McMullin, Steve Mihok, Patrick M. Guerin

**Affiliations:** 1 Tsetse and Trypanosomiasis Research Institute, Tanga, Tanzania; 2 Tsecon Consultants, Nairobi, Kenya; 3 Institute of Biology, University of Neuchâtel, Neuchâtel, Switzerland; 4 Russell, Ontario, Canada; International Centre of Insect Physiology and Ecology, Kenya

## Abstract

**Background:**

Here we set out to standardize long-lasting, visually-attractive devices for *Glossina swynnertoni*, a vector of both human and animal trypanosomiasis in open savannah in Tanzania and Kenya, and in neighbouring conservation areas used by pastoralists. The goal was to determine the most practical device/material that would induce the strongest landing response in *G. swynnertoni* for use in area-wide population suppression of this fly with insecticide-impregnated devices.

**Methods and Findings:**

Trials were conducted in wet and dry seasons in the Serengeti and Maasai Mara to measure the performance of traps and targets of different sizes and colours, with and without chemical baits, at different population densities and under different environmental conditions. Adhesive film was used as a simple enumerator at these remote locations to compare trapping efficiencies of devices. Independent of season or presence of chemical baits, targets in phthalogen blue or turquoise blue cloth with adhesive film were the best devices for capturing G. *swynnertoni* in all situations, catching up to 19 times more flies than pyramidal traps. Baiting with chemicals did not affect the relative performance of devices. Fly landings were two times higher on 1 m^2^ blue-black targets as on pyramidal traps when equivalent areas of both were covered with adhesive film. Landings on 1 m^2^ blue-black targets were compared to those on smaller phthalogen blue 0.5 m^2^ all-blue or blue-black-blue cloth targets, and to landings on all-blue plastic 0.32–0.47 m^2^ leg panels painted in phthalogen blue. These smaller targets and leg panels captured equivalent numbers of *G. swynnertoni* per unit area as bigger targets.

**Conclusions:**

Leg panels and 0.5 m^2^ cloth targets show promise as cost effective devices for management of *G. swynnertoni* as they can be used for both control (insecticide-impregnated cloth) and for sampling (rigid plastic with insect glue or adhesive film) of populations.

## Author Summary

*Glossina swynnertoni* is restricted to open savannah in north-western Tanzania and south-western Kenya, where it is a vector of both human and animal trypanosomiasis in wildlife reserves and in neighbouring conservation areas used by pastoralists. Despite the challenge to minimize disease transmission through effective management of the vector in the presence of abundant wildlife reservoirs, little has been done to test the efficacy of modern tsetse traps or targets for controlling *G. swynnertoni*. We made field tests in the Serengeti and Maasai Mara to determine the most visually-attractive, long-lasting and practical object that induces the strongest landing response in *G. swynnertoni*. Fly landings were twice as high on 1 m^2^ blue-black targets as on pyramidal traps when equivalent areas of these devices were covered with adhesive film. Furthermore, blue leg panels in either cloth or plastic and blue or blue-black-blue cloth targets under half the size of traditional targets captured tsetse at equivalent numbers per unit as the latter. These smaller targets and leg panels show promise as cost-effective devices for management of *G. swynnertoni* populations as they can be used for both control (insecticide-impregnated cloth) and monitoring of this species (rigid plastic with insect glue or adhesive film).

## Introduction

*Glossina swynnertoni* Austen (Diptera, Glossinidae) is restricted to open savannah in north-western Tanzania and south-western Kenya, extending from Tarangire in the south through Manyara to the Serengeti plains, and into the Maasai Mara in the north [1]. Swynnerton [2] found it at 900–1800 m above sea level and considered temperature, humidity, vegetation and the presence of wildlife as the key factors controlling its distribution. It is a vector of both human and animal trypanosomiasis in wildlife reserves and in neighbouring conservation areas used by pastoralists [3]–[6]. The challenge is to minimize disease transmission through effective management of the vector in the presence of abundant wildlife reservoirs, especially in protected areas.

*G. swynnertoni* is in the savannah or *morsitans* group of tsetse. Considerable progress has been made in developing visually-attractive control devices such as traps [7] and insecticide-impregnated targets [8], [9] for this group. However, no comparable effort has been made to develop cost-effective devices for *G. swynnertoni* since initial tests were conducted in Tanzania in 1991–1993 [10]. Presently, local control of this species is being attempted with techniques refined for other species. For example in Kenya, pastoralists deploy insecticide-impregnated targets or apply pyrethroid sprays to livestock [11] in a largely uncoordinated effort at vector control.

Savannah tsetse are attracted to artificial objects of modest size [9] that are conspicuous relative to their immediate environment [12]. Traps and targets of phthalogen blue (peak reflectance at 465 nm) and/or black cloth of about 1 m in dimension are typically effective for this group of tsetse [13]. *G. swynnertoni*, like G. *morsitans* [8], is nevertheless difficult to catch with simple stationary devices, as movement and other subtle visual cues [14] are likely involved in host-seeking behaviour. Vehicle patrols or “fly-rounds” were previously used for sampling *G. swynnertoni* [15], [16], but recent studies now mostly use blue-black cloth traps designed for other tsetse [6]. There have been few comparative tests of the efficacy of modern tsetse traps or targets relative to other methods for collecting *G. swynnertoni* outside of the works of Ndegwa & Mihok [17] and Ndegwa et al. [18]. These studies showed that trap designs other than the S3 trap were relatively inefficient compared to a 1 m^2^ black sticky target. Unlike *Glossina pallidipes* Austen (Diptera, Glossinidae), baiting traps with attractants such as acetone, 1-octen-3-ol, phenols and/or cow urine does not result in large increases in catch [10], [19].

Within the Africa-wide WHO-TDR initiative to develop innovative control strategies for tsetse, we set out to standardize long-lasting, visually-attractive devices for *G. swynnertoni*. The trials were based on existing trap/target/bait technology following a similar experimental approach throughout Africa [20]. Trials were conducted in wet and dry seasons in the Serengeti and Maasai Mara to measure the performance of pyramidal traps and targets in phthalogen blue and various alternatives at different population densities and under different environmental conditions. A simple enumeration method (sticky film) was used at these remote locations to compare trapping efficiencies of devices made of well-characterized colour-fast fabrics (and a blue-painted plastic). The relative performance of devices was also compared with and without chemical baits. Various alternatives were compared to a standard phthalogen blue and black pyramidal trap, which has been the tsetse survey device of choice in Tanzania in recent years [4]. The overall goal was to determine the most practical device/material that would induce the strongest landing response in *G. swynnertoni* for future use in area-wide population suppression of this fly with insecticide-impregnated devices.

## Materials and Methods

### Study sites

Studies were conducted in open *Acacia*-*Commiphora*-*Balanites* dry savannah woodland in pastoral areas near the Maasai Mara National Reserve in Kenya and deep within the neighbouring Serengeti National Park across the border in Tanzania. Wild hosts remain abundant in the Serengeti but have declined considerably in the Mara in the last few decades [21]. Livestock were present only in the Mara.

In the Serengeti, three sets of studies took place at Death Valley 2°19′51″ S, 34°49′60″E at an altitude of 1548 m near Seronera. A first set of experiments was conducted in 2009 in the wet season (July) and repeated at the same sites in the dry season (October). In 2010 and 2012, a second and third series were conducted at the same location, both at the start of the dry season (September). *G. pallidipes* was also present in scattered evergreen thickets. Findings for this species are reported where captures were adequate for analysis.

In the Mara, studies were undertaken at the Olarro hills 1°25′45.4″S, 35°35′0.9″E at an altitude of 1910 m, and at the Nyonorri hills at 1°27′18.9″S, 35°33′43.5″ E at an altitude of 1877 m. Unbaited and baited trials were conducted at these locations separately in the nominal wet season (June), and then repeated only at one location (Nyonorri) in the subsequent dry season (November) of 2009. The shift in locations was necessitated by the unanticipated use of pyrethroid spray-ons by pastoralists at Olarro.

Habitats have been extensively altered by human activities in pastoral areas near the Mara. Hence, *G. swynnertoni* is mainly found in remnant woodlands along hillsides that receive moisture from the highlands all year-round. A severe drought was underway during these tests; hence wild hosts were present only in the nominal “wet season”. Vegetation cover was particularly sparse during all seasons in the Mara due to the drought. Cover in the Serengeti was not affected as much, and hence wet and dry season contrasts were more typical of natural climatic cycles in Tanzania.

### Catching devices, materials and baits

In 2009, three catching devices were tested: standard cloth pyramidal traps [22], rectangular cloth targets, and smaller all-blue “leg” panels [23]. The dimensions and design of the targets and leg panels were chosen to reflect current practices in East Africa and are summarised in Table 1. Devices were set in the open, 30 cm off the ground; vegetation was removed from within a few metres of each site. The targets in Kenya were 1.5 m^2^ (1.5 m wide by 1 m high) divided vertically into equal rectangles of blue and black cloth [24]. In Tanzania, the targets were the same size but were divided vertically into three equal rectangles of blue-black-blue [25]. Leg panels in Kenya were made of blue cloth with a surface area of 0.32 m^2^ (65 cm wide by 46 cm high for the upper “torso”, plus two “legs” 15 cm high by 8 cm wide). Two kinds of slightly larger leg panels were tested in Tanzania. One was 0.47 m^2^ blue cloth (70 by 64 cm plus 14 by 9 cm legs) and the other was 0.45 m^2^ blue-painted plastic (90 by 45 cm plus 15 by 15 cm legs). The plastic was 3–4 mm thick and was painted glossy phthalogen blue. All targets were mounted on supports that allowed for limited rotational movement in the wind. The wet season trial in Tanzania occurred under particularly windy conditions.

**Table 1:** Trapping devices used in experiments and their surface areas.

Object	Type and colour combination	Size	Sticky surface area
**2009 & 2010 trials**			
**Target**	blue/black 1∶1	1×1 m	2 m^2^
	Kenyan** blue/black 1∶1	1.5×1 m	3 m^2^
	Tanzanian** blue/black/blue 1∶1∶1	1.5 m×1 m	#1.8 m^2^
	transparent adhesive tape	1×1 m	1 m^2^
**Leg panel**	cloth, Ky all blue	0.65 m×0.46 m	0.64 m^2^
	cloth, Tz all blue	0.70 m×0.64 m	0.94 m^2^
	plastic, Tz all blue	0.90×0.45 m	0.90 m^2^
**Pyramidal trap**	standard blue/black 1∶1	-	2 m^2^ (2010 trials only)
**2012 trials**			
**1 m ^2^ target**	blue/black 1∶1	1×1 m	2 m^2^
	blue/black/blue 1∶1.1	1×1 m	2 m^2^
**0.5 m^2^ oblong. target**	blue/black/blue 1∶1.1	0.9 m×0.55 m	1 m^2^
	all-blue	0.9 m×0.55 m	1 m^2^
**0.25 m^2^ target**	blue/black/blue 1∶1∶1	0.5×0.5 m	0.5 m^2^
	all-blue	0.5×0.5 m	0.5 m^2^

# Only the lower 60 cm of the Tanzanian targets was covered with adhesive film.

** Referred to in the text as local target (standard phthalogen blue cotton).

Two blue fabrics were tested: C180 Azur 623 phthalogen blue 100% cotton (180 g/m^2^, TDV, Laval, France) with a reflectance peak at 460 nm as measured with a Datacolor Check Spectrophotometer (Datacolor AG, Dietlikon, Switzerland), referred to hereafter as standard blue cotton, and turquoise blue 65% polyester/35% viscose (234 g/m^2^, Q10067 Sunflag, Nairobi, Kenya) with a peak at 480 nm. The phthalogen blue paint on the plastic leg panel had a peak of 460 nm. A 100% polyester black (225 g/m^2^, Q15093 Sunflag, Nairobi) was used for all devices in all trials described here. To monitor the number of tsetse landing on cloth targets and leg panels, one-sided adhesive film (30 cm wide rolls, Rentokil FE45, UK) was stitched with thread to both sides of the trapping devices. However, in 2009 in Tanzania only the lower 60 cm of the targets was covered. Plastic leg panels in Tanzania were coated with a non-setting shiny glue (Temoocid, Kollant, Italy). Transmittance spectra for both adhesives are compared to polybutene in Figure 4.4 in IAEA TECDOC 1373 [26]. All of these adhesives are highly transparent in the visible spectrum, but Rentokil film absorbs significantly in the ultraviolet (<400 nm).

In 2010, two supplementary trials were conducted in Tanzania to enumerate flies landing on pyramidal traps compared to 1 m^2^ square targets, divided vertically into equal parts of blue and black material (referred to hereafter as the standard target). For this, adhesive film was also attached to the blue-black fabric of the pyramidal traps to enumerate flies that land but may not be captured. In an additional test only 1×1 m squares of adhesive film on its own (i.e. without targets as a backdrop) were compared to 1 m^2^ square targets (with equal parts of blue and black material) covered with adhesive film to ascertain whether the adhesive film in itself was attractive. A further set of trials was conducted in Tanzania in 2012 to compare six different two-dimensional cloth targets to evaluate the influence of size, shape and colour combination on fly landing rates. The six devices were: two types of 1 m^2^ square targets, one divided vertically into equal parts of blue and black material, the other divided vertically into three equal parts of blue-black-blue; two types of 0.5 m^2^ targets (0.9 m×0.55 m), one divided vertically into equal parts of blue-black-blue and the other all blue, both set up horizontally; and two types of 0.25 m^2^ square targets (0.5 m×0.5 m) one divided vertically into equal parts of blue-black-blue, the other all blue (see Table 1).

An 1∶4∶8 mixture of 3-n-propylphenol (P), 1-octen-3-ol (O), and p-cresol (C) (Ubichem research LTD, Budapest, Hungary with a global purity of up to 98%) was used as an attractant for experiments comparing baited devices based on general efficacy for several tsetse species [26]. Sachets made of 500 gauge/0.125 mm polyethylene containing 3 g of the mixture were placed below the catching devices, 10 cm above the ground, next to a 250 ml bottle buried in soil up to the shoulders containing acetone (A) with a 2 mm aperture in the stopper. This combination of chemicals is termed the POCA bait.

### Experimental design

#### Testing trapping devices and blue materials

To assess which was the best catching device and the most attractive blue material in the wet and dry seasons at each location, a six-day experiment was carried out to compare six devices in a 6×6 Latin square design of days×sites×treatments, with 3 simultaneous replicates. Trapping positions were always >100 m apart and flies from each device were counted and sexed after 24 hours at each position. The six devices and blue materials tested were: pyramidal traps in standard blue cotton and turquoise blue polyester/viscose; local targets in standard blue cotton and turquoise blue polyester/viscose; leg panels in standard blue cotton (both countries) and turquoise blue polyester/viscose (Kenya only) or plastic leg panels covered with phthalogen blue paint (Tanzania only). The 6 device experiment was repeated using the POCA bait, after the unbaited trials, in the same general area with trapping positions >200 m apart. Flies from each device were counted after 24 hours at each position. The objective was to determine whether baiting changed the performance ranking of the devices/materials.

#### Landing on trapping devices

To assess the efficiency of 3-d traps versus 2-d targets as landing devices, catches in pyramidal traps with adhesive film on the blue-black fabric were compared to 1 m^2^ blue-black (50∶50) targets covered with adhesive film in 2010. All catching devices were made of standard phthalogen blue cotton and black polyester. Flies caught in the cage of the traps were not included in the total for this comparison. The surface area of adhesive film was the same on both devices, i.e. 2 m^2^. Traps without attached adhesive film were included as controls to estimate trapping efficiency of the pyramidal device. A 3-day experiment was carried out to compare the three devices in a 3×3 Latin square of days×sites×treatments in four replicates. The trapping positions were always >100 m apart and flies of each sex from each device were counted after 24 hours at each position.

#### Landing on targets of different size, shape and colour

To assess the influence of size, shape and colour on fly landings, six target types (two of 1 m^2^ square, two 0.5 m^2^ horizontal oblongs and two 0.25 m^2^ square targets; see Table 1) were compared in 2012 in a 6×6 Latin square design experiment of days×sites×treatments, with 3 simultaneous replicates. All targets were made of standard phthalogen blue cotton and black polyester. Target positions were set up and fly counts were made as in previous experiments.

**Figure 1:**
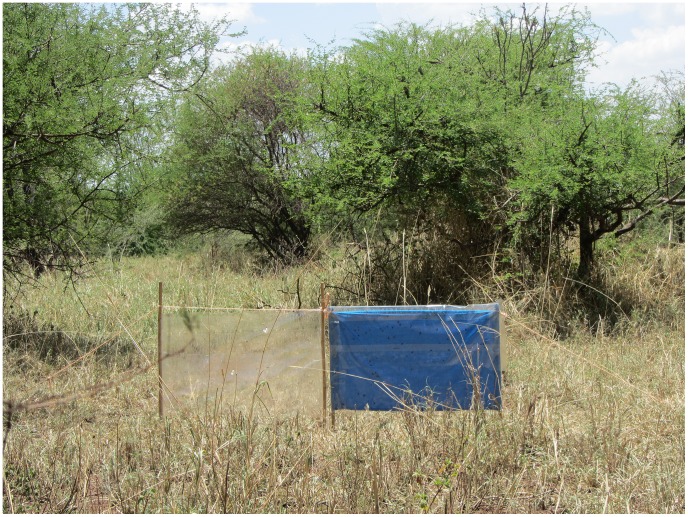
Blue cotton target with adhesive film and adjoining adhesive film target in Serengeti tsetse habitat.

To investigate the efficiency with which 0.5 m^2^ targets capture tsetse, a fully randomized trial was made in 2012 with three replicates of oblong sticky targets in blue-black-blue and all blue (Table 1) were each flanked with an adjoining transparent adhesive film target of the same shape and size (sticky on only one side; Figure 1). The aim was to estimate what proportion of flies attracted to the targets circle the device.

#### Testing adhesive film

To assess whether the adhesive film on its own was attractive to tsetse and could affect the catching device, a comparison was made between catches of tsetse attracted to a stationary 1 m^2^ cloth target of standard phthalogen blue cotton and black polyester (50∶50) with adhesive film applied on both sides with a 1 m^2^ square of adhesive film alone (sticky on one side only, minimal supports). The two devices were orientated E–W, and a 4-day experiment was conducted following a 2×2 Latin square design of days×sites×treatments in four simultaneous replicates. The trapping positions were always >100 m apart and flies from each device were counted and sexed after 24 hours at each position

#### Normalizing fly catches

Area-wide population suppression involves consideration of the cost-effectiveness of materials versus deployment and maintenance. Hence, it was important to also quantify catches normalized for the size of each trapping device in the main series of experiments. To derive an empirical adjustment factor for the fact that in 2009, only the bottom 60 cm of the targets in Tanzania was covered with adhesive film, we recorded the heights of flies landing on the blue-black targets in an indicative experiment to test if a standard blue-black target (1 m^2^, Table 1) would catch as many flies (covered on both sides with adhesive film) as a local blue/black Kenyan target (1.5 m^2^, Table 1).

### Statistical analysis

In all trials randomization was set up using design.lsd in the package *agricolae* [27], R version 2.13.0 [28]. Data were analysed using a linear model in R version 2.13.0 [28], including the following additional packages: MASS [29] and multcomp [30]. Analysis was performed on log (x+1) transformed data including day and position as ordering parameters and Tukey contrasts were calculated to compare treatments. The Wilcoxon paired test was used to compare fly landings on the blue and black portions of targets and to compare catches on the transparent film versus the blue-black target. Unless otherwise specified, results are presented as detransformed means. *G. pallidipes* is not mentioned where captures were too low for meaningful analysis.

## Results

### Performance of unbaited trapping devices

**Figure 2:**
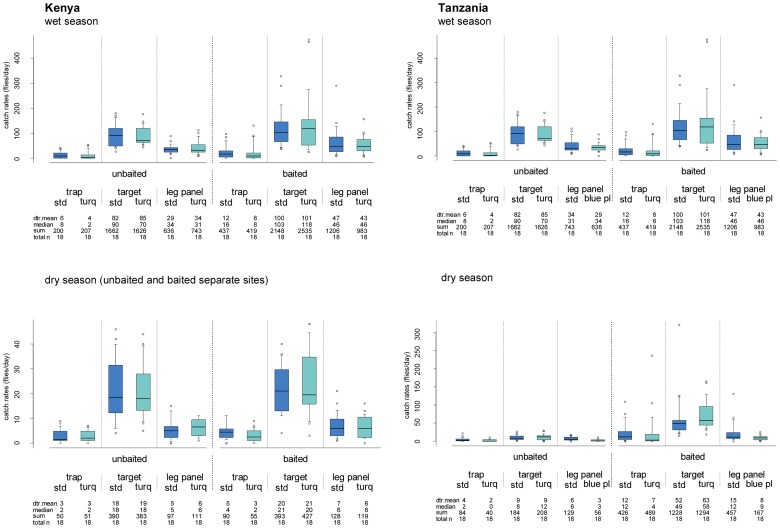
Detransformed daily trapping rates for *G. swynnertoni* by unbaited and POCA-baited visual devices. **trap** pyramidal trap, **target** 1.5 m^2^ target, **leg panel** local panels (0.47 m^2^ Tanzania, 0.32 m^2^ Kenya), **std** standard phthalogen blue, **turq** turquoise blue, **blue pl** blue-painted plastic (0.45 m^2^), **unbaited** no baits, **baited** baited with POCA, **dtr**. **mean** detransformed mean. POCA is a 1∶4∶8 mixture of 3-n-propylphenol (P), 1-octen-3-ol (O), and p-cresol (C) released from a polyethylene sachet and acetone (A) released from a bottle. The limits of the boxes indicate the twenty-fifth and seventy-fifth percentiles; the solid line in the box is the median; the capped bars indicate the tenth and the ninetieth percentiles, and data points outside these limits are plotted as circles.

When unbaited, both types of blue-black targets (Kenyan and Tanzanian) covered with adhesive film were the best devices for *G. swynnertoni*. In both countries and irrespective of season or fabric, sticky targets in the unbaited trials captured more *G. swynnertoni* than pyramidal traps (P≤0.001, Table 2 and Figure 2). Catches were 2.4–6.7 times higher in three of the trials, and nearly 20 times higher in one trial (wet season, Tanzania). Targets covered with adhesive film also out-performed the smaller all-blue leg panels (all types and regardless of adhesive), capturing 2.2–3.7 times more flies in Kenya (P≤0.01, Table 2) and 1.5–2.8 times more flies in Tanzania (P<0.05 for the plastic leg panel, not significant for the cloth leg panel, Table 2). The leg panels similarly captured more flies than the pyramidal traps in Kenya (P<0.05, wet and dry season, Table 2) and Tanzania (P≤0.001, wet season, not significant P>0.05 in the dry season, Table 2). There was no difference between the performance of any of the same devices made from the different blue materials (P>0.05; Table 2 and Figure 2), and sex ratios were similar on the different devices.

**Table 2:** Catches* of *G. swynnertoni* with unbaited and POCA-baited trapping devices made of different blue materials.

		Kenya				Tanzania			
Device	Blue material	Wet season		Dry season		Wet season		Dry season	
		unbaited	POCA	unbaited	POCA	unbaited	POCA	unbaited	POCA
Pyramidal	standard	*1.5^a^*	*7.9^a^*	*3.0^a^*	*4.9^a^*	*6.1^a^*	*11.6^a^*	*3.8^ab^*	*12.3^a^*
	turquoise	*1.6^a^*	*8.0^a^*	*2.9^a^*	*3.1^c^*	*4.3^a^*	*8.0^a^*	*2.1^a^*	*6.7^a^*
Target	standard	*7.8^b^*	*49.3^b^*	*18.5^b^*	*20.3^b^*	*81.5^b^*	*99.6^b^*	*9.1^c^*	*51.5^b^*
	turquoise	*7.7^b^*	*44.1^b^*	*19.3^b^*	*20.7^b^*	*84.6^b^*	*101.2^b^*	*9.2^c^*	*63.0^b^*
Leg panel	standard	*3.5^c^*	*15.3^c^*	*4.9^c^*	*6.5^a^*	*34.0^bc^*	*46.6^bc^*	*6.2^bc^*	*14.8^a^*
	turquoise	*3.1^c^*	*10.1a^c^*	*6.2^c^*	*6.1^a^*				
	plastic					*28.5^c^*	*42.7^bc^*	*3.1^ab^*	*7.8^a^*

* Detransformed mean daily catches. ANOVA indicates significant differences between treatments within each column; means followed by different letters are significantly different (Tukey post hoc test, P<0.05).

### Performance of POCA-baited trapping devices

The relative rankings of the POCA-baited devices were very similar to those of unbaited devices for *G. swynnertoni*. As before, the sticky targets greatly outperformed the pyramidal trap, with the largest difference in catch in the wet season in Tanzania (P≤0.001, Table 2 and Figure 2). Catches were 5.5–6.7 times higher in three of the trials and up to 12.7 times higher in the wet season in Tanzania. In Kenya, the baited target captured 3.2–4.2 times more flies than the smaller leg panels in both seasons (P≤0.001, Table 2), and an average of 5.6 times more in the dry season in Tanzania. In contrast, in the wet season in Tanzania, the catches of the target were on average 2.2 times higher than on leg panels and this was not significantly higher on either the cloth or plastic leg panels (P>0.05, Table 2). The baited leg panels consistently caught more flies than baited pyramidal traps, but not all contrasts were significant; In Kenya, where just cloth leg panels were tested, only two out of eight comparisons were significant (P≤0.05, Table 2, the standard blue leg panel compared with both pyramidal traps in the wet season and both leg panels compared to the turquoise blue pyramidal in the dry season). In Tanzania, leg panels in both cloth and plastic caught significantly more flies than pyramidal traps in the wet season (4.6 times, P≤0.001, Table 2), but there was no difference amongst all four devices in the dry season (P>0.05, Table 2). As in the unbaited trials, there was no difference between the performance of any of the same devices (trap, target, leg panel) made from different blue materials (P>0.05, Table 2 and Figure 2), and sex ratios were similar on the different devices. Baited devices were tested shortly after unbaited devices for logistical reasons, hence differences in catches across the two sets of experiments of the same devices have not been interpreted.

### Landing on trapping devices

**Figure 3:**
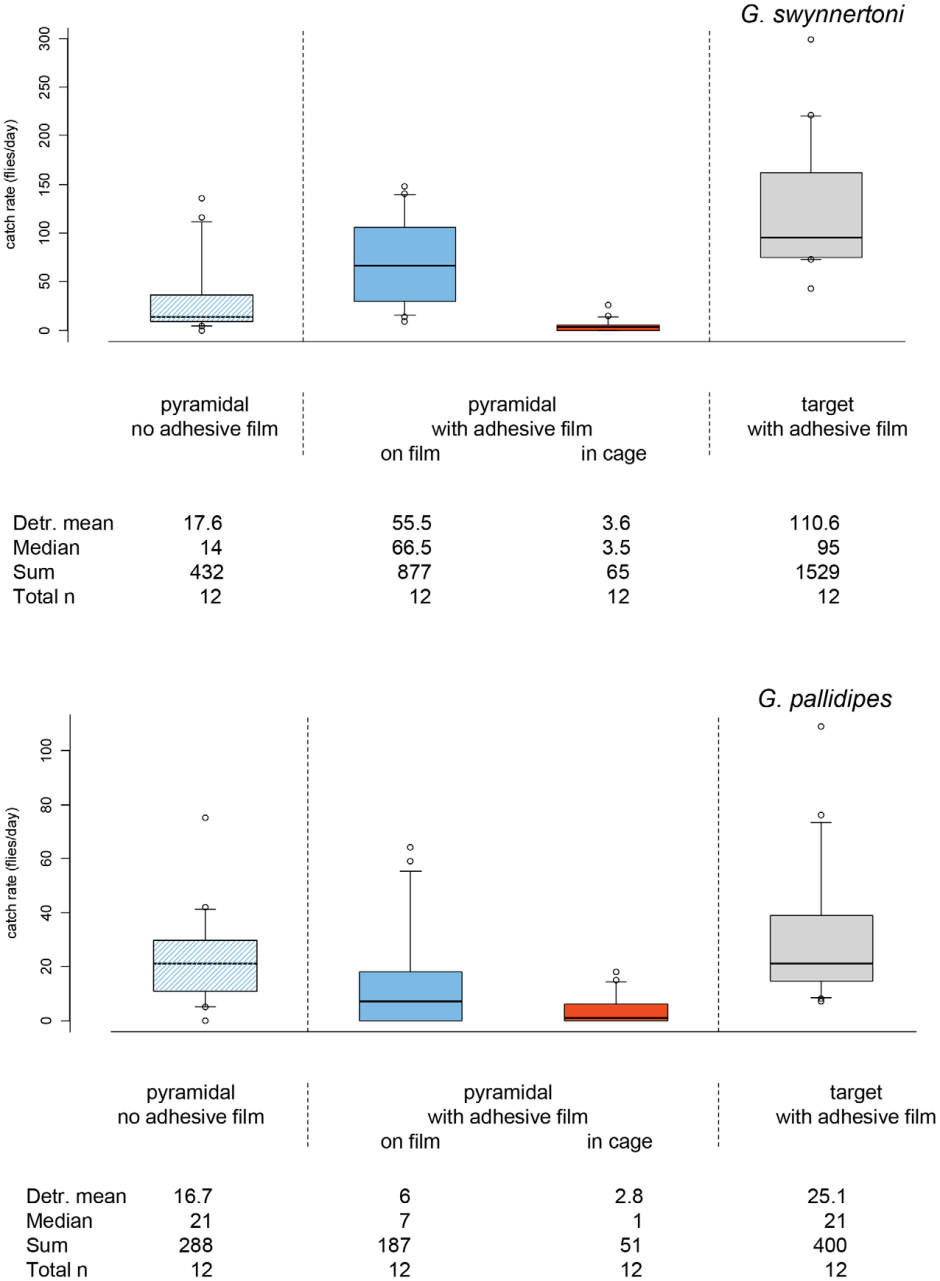
Daily catches of *G. swynnertoni* and *G. pallidipes* by devices with and without adhesive film. **pyramidal** pyramidal trap; **target** blue-black 1 m^2^ target. The target and the cloth portions of traps were covered with adhesive film to compare the propensity of flies to land on the different devices. Catch rates of traps are divided into fly catches on the cloth part and those trapped in the cage of the trap. The limits of the boxes indicate the twenty-fifth and seventy-fifth percentiles, the solid line in the box is the median, the capped bars indicate the tenth and the ninetieth percentiles, and data points outside these limits are plotted as circles.

A 2-d blue-black 1 m^2^ target with attached adhesive film induced more *G. swynnertoni* to land relative to a 3-d pyramidal trap with its blue-black surfaces covered with the same surface area of film (Figure 3). Twice the number of flies landed on the target relative to the pyramidal trap (110.6/55.5, P<0.05; Figure 3), and six times more flies landed on the target than were caught in a control trap without film (110.6/17.6, P<0.05; Figure 3). For *G. pallidipes*, 3.3 times more flies landed on the target than the pyramidal trap covered with adhesive film (25.1/6.0, P<0.05; Figure 3), and 1.5 times more landed on the target than in the control trap without adhesive film (25.1/16.7, P>0.05; Figure 3). Sex ratios were similar on the three devices for both species.

**Figure 4:**
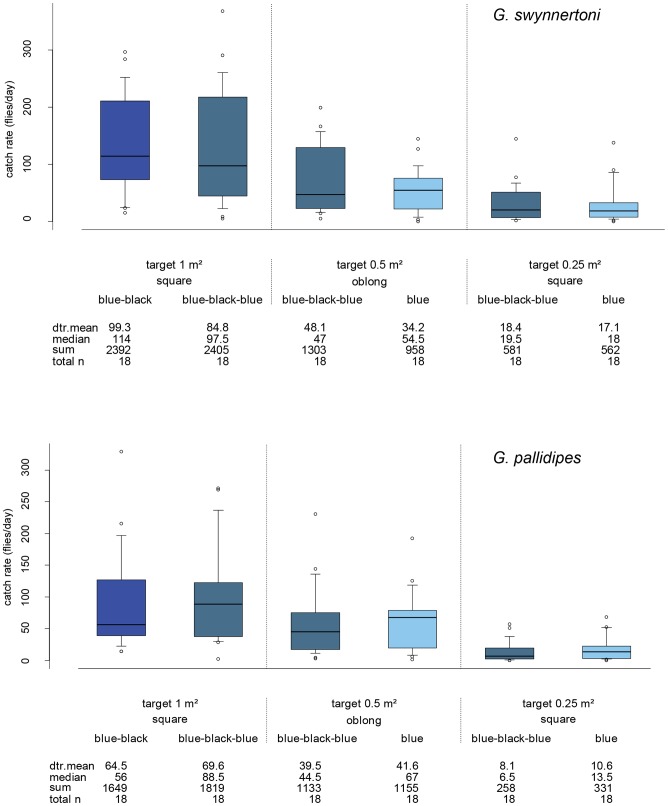
Daily catches of *G. swynnertoni* and *G. pallidipes* with different target types. Targets were all blue, or of equal vertical rectangles of blue and black or blue-black-blue material. Devices were covered with adhesive film to compare the propensity of flies to land on the different target types. The limits of the boxes indicate the twenty-fifth and seventy-fifth percentiles; the solid line in the box is the median; the capped bars indicate the tenth and the ninetieth percentiles, and data points outside these limits are plotted as circles.

### Efficiency of the pyramidal trap

Trap efficiency, i.e. the proportion of flies caught in the trap cage of those that approach at close range, was estimated by dividing the mean daily catch in the cage of the unaltered pyramidal trap by the mean daily catch of the trap with adhesive film on the cloth, i.e. summing flies caught on adhesive film and in the cage. Efficiency for *G. swynnertoni* was 30% (17.6/(55.5+3.6)×100, Figure 3). Very few flies were caught in the cage of traps with adhesive film (6%), suggesting that few flies are caught without first landing on the blue-black cloth. Trap efficiency for *G. pallidipes* could not be estimated as the trap with adhesive film caught fewer flies than the trap without film.

### Testing adhesive film

The 1 m^2^ target of adhesive film on its own (unbaited) caught very few tsetse of either species compared to the cloth target covered with adhesive film. This target caught 2% of the detransformed mean daily catch of *G. swynnertoni* on the cloth target (2.9/119.1, P≤0.05), and 6% of the detransformed mean daily catch of *G. pallidipes* (1.3/21.3, P≤0.05). Note that the sticky surface area of the cloth targets was twice that of the stand-alone adhesive film target.

### Performance of leg panels as landing devices

Catches were low in the indicative experiment to test if a standard 1 m^2^ blue-black target would catch as many flies as a local blue/black 1.5 m^2^ Kenyan target as pastoralists were attempting to reduce tsetse in the study area in Kenya when the experiments were conducted and vegetation was also being heavily-grazed. The 1.5 m^2^ target caught a mean of 5.5 flies versus 3.0 flies on the standard 1.0 m^2^ target (fly counts not statistically analysed). However, when landing heights were tallied, 101 of 132 *G. swynnertoni* (77%) landed on the bottom 60 cm of the targets. This information was used to obtain an indicative normalized catch per m^2^ for the Tanzanian targets in the main experiments of 2009 where only the bottom 60 cm was covered. The detransformed mean catch on adhesive film was multiplied by 1.30 (1/0.77) for flies that could have landed on the upper 40 cm of the target without them landing on the lower 60 cm with adhesive (an assumption, i.e. a maximum estimate), divided by 3 m^2^. All other detransformed mean catches were normalized only for the size of the trapping device (Table 1) as in every other case the entire device was covered in adhesive film. Based on this logic, detransformed mean catches per m^2^ of total surface area of Kenyan cloth leg panels averaged 1.5 times those of the Kenyan local target (range 1.0–2.1 times, Table 3), with similar trends among turquoise and standard phthalogen blue cloth. After adjusting for the partial adhesive coverage of Tanzanian targets, detransformed mean catches on Tanzanian leg panels averaged 1.0 times those of the Tanzanian local target (range 0.4–1.7 times, Table 3), with similar trends for turquoise and standard blue cloth, or blue-painted plastic. Note that the leg panels in Kenya were smaller than in Tanzania, and the Kenyan target, although of the same size as in Tanzania, had a different configuration of blue - black. Considering the high performance of the leg panels relative to the, on average, 3.5 times bigger targets, we conducted an extra experiment to assess the effect of target size, shape and colour on the landing responses of flies.

**Table 3:** Catches* and catch indices** for *G. swynnertoni* normalized to an equal area for each device.

Country - season		STD blue-black target	STD blue leg panel		TURQ blue leg panell		Blue-painted plastic leg panel	
		flies/m^2^	flies/m^2^	catch index	flies/m^2^	catch index	flies/m^2^	catch index
**Kenya - wet**	Unbaited	2.6	5.5	**2.1**	4.8	**1.9**		
	Baited	16.5	23.9	**1.5**	15.8	**1.0**		
**Kenya - dry**	Unbaited	6.2	7.7	**1.2**	9.7	**1.6**		
	Baited	6.8	10.2	**1.5**	9.5	**1.4**		
**Tanzania - wet**	Unbaited	35.3	36.2	**1.0**			31.6	**0.9**
	Baited	43.2	49.6	**1.2**			47.4	**1.1**
**Tanzania - dry**	Unbaited	4.0	6.6	**1.7**			3.4	**0.9**
	Baited	22.3	15.8	**0.7**			8.7	**0.4**

* Detransformed mean daily catches.

** Catch indices are relative to the performance of a conventional blue-black cloth targets (left column).

**STD** Standard phthalogen blue cotton.

**TURQ** Turquoise blue viscose/polyester.

### Optimal target colour and size, and target efficiency

The 1 m^2^ targets in blue-black (standard) and blue-black-blue equal sized vertical stripes (Tanzanian style) both caught very similar numbers of both *G. swynnertoni* and *G. pallidipes* which suggests that there is no difference between the two designs for inducing landing (P>0.05; Figure 4). The daily landing rate by flies on the blue-black-blue 0.5 m^2^ oblong targets was higher than the all blue targets of similar size for *G. swynnertoni* (48.1 and 34.2 flies/day, respectively; Figure 4), but this difference is not significant (P>0.05). The landing rate by *G. pallidipes* on the two 0.5 m^2^ oblong target types was very similar (P>0.05; Figure 4). Likewise, there was little difference between the daily landing rates by flies of either species on the blue-black-blue and all blue smaller 0.25 m^2^ square targets (P>0.05; Figure 4).

The linear model indicates that beside the ordering factors of target position and experimental day, size is the only significant parameter retained (P<0.001), i.e. neither colour pattern (blue-black-blue, blue-black or all blue) nor shape (oblong or square) significantly affects landings by *G. swynnertoni* or *G. pallidipes*. Fly landings on the 0.5 m^2^ targets were reduced to 44% of the 1 m^2^ targets for *G. swynnertoni* and to 60% for *G. pallidipes* (P<0.01 for both), and to 19% and 14%, respectively, on the 0.25 m^2^ target compared to the 1 m^2^ targets (P<0.01 for both; Table 4). Reducing target size from 0.5 m^2^ to 0.25 m^2^ caused landings to be reduced to 44% for *G. swynnertoni* but to only 23% for *G. pallidipes* (P<0.01 for both; Table 4). All percentages were calculated by de-transforming the coefficients from the linear model and are very similar to the catch indices calculated from the detransformed means in Table 4. Analysis of fly landings on the blue-black-blue targets alone also retained size as a significant factor (P<0.001).

**Table 4:** Catch indices* for targets corrected to a uniform area of 1 m^2^.

	*G. swynnertoni*				*G. pallidipes*			
Target type	Actual catch		Catch normalised to 1 m^2^		Actual catch		Catch normalised to 1 m^2^	
	Flies/m^2^	Catch index	Flies/m^2^	Catch index	Flies/m^2^	Catch index	Flies/m^2^	Catch index
**1×1 m** square blue/black	99.29	**1.0**	99.29	**1.0**	64.53	**1.00**	64.53	**1.00**
**1×1 m** square blue/black/black	84.79	**0.85**	84.76	**0.85**	69.60	**1.08**	69.60	**1.08**
**0.55×0.9 m** oblong blue/black/blue	48.12	**0.48**	96.42	**0.97**	39.54	**0.61**	79.08	**1.23**
**0.55×0.9 m** oblong all blue	34.22	**0.34**	68.44	**0.69**	41.62	**0.64**	83.24	**1.29**
**0.5×0.5 m** square blue/black/blue	18.42	**0.19**	73.64	**0.74**	8.06	**0.12**	32.24	**0.50**
**0.5×0.5 m** square all blue	17.12	**0.17**	68.48	**0.69**	10.60	**0.16**	42.40	**0.66**

* All indices calculated against a standard blue-black 1 m^2^ target (using detransformed mean daily catches).

When the daily landing rates are corrected to an equal target size of 1 m^2^, landings on the 0.5 m^2^ oblong blue-black-blue targets are nearly the same as on the standard blue-black targets for *G. swynnertoni* and *G. pallidipes* (Table 4). These corrected landing rates also indicate that landings on the best performing 0.25 m^2^ square target are 74% of those on the standard 1 m^2^ target for *G. swynnertoni* but only 66% for *G. pallidipes* (Table 4). For *G. swynnertoni*, the ratio of flies landing on the blue and black portions of the bicolour targets was very close to 50∶50, irrespective of the area of each colour, with females showing a slight preference for the black portion (55%) and males a slight preference for the blue (55%). In contrast, in *G. pallidipes*, both sexes showed a strong preference for landing on the blue portion of targets (85%; P<0.001).

In the experiment with adjoining adhesive film targets placed next to the 0.5 m^2^ oblong targets (Figure 1), only 1% of the total *G. swynnertoni* catch on the blue-black-blue target was caught by the adjacent transparent target (6 of 464 flies) and the proportion was 6% for the all blue target (15 of 256 flies). In the case of *G. pallidipes*, only 2% of the total catch on both coloured target types was made on the adjacent transparent target (11 of 457 flies for the blue-black blue target and 5 of 288 flies for the all blue target).

## Discussion

This study shows that independent of season or presence of chemical baits, targets in phthalogen blue or turquoise blue cloth and covered with adhesive film proved the best devices for capturing G. *swynnertoni* in all situations, catching up to 19 times more flies than pyramidal traps. Baiting with chemicals did not affect the relative performance of devices. When equivalent areas of targets and pyramidal traps were covered with adhesive film, fly landings were twice as high on 1 m^2^ blue-black targets as on the pyramidal traps. When landings on 1 m^2^ blue-black targets were compared to those on smaller 0.5 m^2^ all-blue or blue-black-blue cloth targets and to landings on all-blue plastic 0.32–0.47 m^2^ leg panels, the smaller targets and leg panels captured equivalent numbers of *G. swynnertoni* and *G. pallidipes* per unit area as bigger targets.

### Comparison of trapping devices and fabric types

In both Tanzania and Kenya, and independent of season or the presence of baits, targets covered with adhesive film were the best trapping devices for *G. swynnertoni* in all situations, catching over 19 times more tsetse than the pyramidal traps. When not baited, large blue-black targets captured 1.5 to 3.7 times more tsetse than much smaller leg panels. Blue leg panels made of either phthalogen blue, turquoise cloth or phthalogen blue-painted plastic nevertheless captured more tsetse than pyramidal traps, or at worst, equivalent numbers. Both targets and leg panels were particularly effective relative to pyramidal traps during the wet season in Tanzania. Of all the experiments, the wet season trials in the Serengeti represented the greatest challenge in terms of attracting flies to artificial devices during peak vegetation cover [31].

Small leg panels that deviate from large square or oblong blue-black fabric targets, [32], [20], [33], were tested as an alternative for *G. swynnertoni* based on their efficacy for sampling *G. austeni* in Zanzibar [34]. Indeed the performance of leg panels covered with insect glue was remarkably high in capturing *G. swynnertoni*. Leg panels were 21% of the surface of targets in Kenya and 30% of the surface in Tanzania, but per unit area, captured 1.5 times more flies than the targets in Kenya and the equivalent number to targets in Tanzania. These results with leg panels in 2009 were confirmed in Tanzania in 2012 when similarly sized 0.5 m^2^ oblong blue-black-blue cloth targets covered with adhesive tape induced landings per unit area at nearly the same level as on the 1 m^2^ targets for *G. swynnertoni*. The potential cost-effectiveness of small targets for different tsetse has been demonstrated only very recently for a few tsetse species [32], [35]. This success with smaller all-blue leg panels and all-blue or blue-black-blue cloth targets as landing devices for *G. swynnertoni* stands out relative to poor results for small all-black targets for two other savannah species *G. pallidipes* and *G. morsitans morsitans* in Zimbabwe [9]. However, earlier results have shown that all blue and blue-black targets perform better than all-black targets for *G. pallidipes* [25]. Indeed, the 0.5 m^2^ oblong target in blue-black-blue or all blue cloth induced landings per unit area at nearly the same level as on the 1 m^2^ targets for *G. pallidipes* in our trials. This may be related to the predominance of blue in the 0.5 m^2^ targets tested here, but concurs with earlier findings where doubling the target size doubles the catch for *G. pallidipes* [25].

*G. swynnertoni* lives in very open and often windy habitats where visual cues (including colour) may be more important than host odours; this is also manifested in terms of its well-known attraction to large, moving objects [16]. Regardless of this, small blue leg panels or targets of approximately 0.5 m^2^ in size clearly show promise for trapping *G. swynnertoni*, particularly as wind damage can be a problem with the larger local targets at many sites. However, 50% of flies captured landed on the black portion of all the targets tested, even those with only one third of the surface area in black. It would therefore be advisable to maintain a black element in visual devices targeting this species, as the presence of adhesive film used in these experiments has been shown to significantly reduce the landing rate on the black section of targets by *G. tachinoides* and G. *palpalis gambiensis* [20]. It is therefore likely that the proportion of *G. swynnertoni* landing on the black would be higher on unmodified targets. Similar catches with cloth and plastic leg panels also indicate that this strategy can be used for both control (insecticide-impregnated cloth) and sampling of this species (rigid plastic with insect glue or adhesive film). Considering the fall off in landings per unit area on the very small 0.25 m^2^ targets, it would be inadvisable to employ targets much smaller than 0.5 m^2^ for control programmes.

There was no difference between the performance of any of the same devices made from the different blue materials between seasons and locations. The two blue fabrics chosen for these experiments (phthalogen blue cotton and turquoise blue polyester/viscose) were manufactured with only minor differences in fabric texture and with slight but clear differences in blue-green colour. These fabrics, and the equivalent blue paint used on plastic leg panels [36], performed equally well in targets/leg panels and traps under diverse conditions for *G. swynnertoni*. These results agree with findings for the same fabrics tested in similar devices for several tsetse species in West Africa [20]. Phthalogen blue cotton cloth has been used for about 30 years in tsetse sampling and control, and is the standard against which all other blues should be compared for attractive properties [13]. It has the maximum colour fastness possible for a pure blue fabric due the formation of copper phthalocyanine (Pigment Blue 15) *in situ* through a unique dyeing process but now remains in limited production in just a few countries. This has resulted in the *ad hoc* use of several alternative blue fabrics in tsetse control, some of which are less than optimal for attracting tsetse [37]. Hence, it has become important to develop appropriate fabrics that can be produced locally with non-proprietary methods. The turquoise blue fabric, produced in Kenya by Sunflag for these experiments using generic dyes and processes, performed well in our studies. This clearly shows that it is possible to produce a deep turquoise that can be used as a practical alternative to phthalogen blue, as suggested by Mihok et al., [38]. Their suitability in control devices is currently being investigated in terms of optimising colour-fastness and insecticide-retaining qualities.

### Performance of targets versus traps as landing devices

This study provides a comparison of the efficacy of several target designs relative to the most common simple trap (pyramidal) in current use for the savannah tsetse *G. swynnertoni*. For a standard 1 m^2^ blue-black target, catches were twice as high as on the equivalent area of a pyramidal trap for *G. swynnertoni*, and three times higher for *G. pallidipes*. The adhesive film used to enumerate tsetse here (and also in Rayaisse et al., [20]) was found to be unattractive when used alone. The fabrication of insecticide-impregnated cloth targets has obvious practical advantages over traps for area-wide population suppression programmes. The potential cost effectiveness of using target-type devices for controlling *G. swynnertoni* is highlighted in this study by the efficacy with which leg panels trap the species. Per unit area, leg panels and 0.5 m^2^ oblong targets were as effective as the two local styles of bigger targets in common use in Kenya and Tanzania. The efficacy of smaller 2-d devices for capturing *G. swynnertoni* follows a pattern recently demonstrated for a range of riverine spp. [32], [33], [35], [39]. Evidently, simple blue-black-blue or all-blue targets and all-blue leg panels of equivalent size are clearly effective in providing adequate visual stimuli to attract *G. swynnertoni* to land, the key behaviour that underlies the principle of insecticide-impregnated control devices and they are also less prone to wind damage because of their smaller size.

### Effect of the POCA bait on trap and target performance

Pyramidal trap entry/retention did not appear to be improved by baiting traps with POCA, i.e. baited targets still caught far more tsetse than baited pyramidal traps. As the baited and unbaited trials were sequential, they could not be compared directly. Nevertheless, our results are consistent with previous failures to substantially improve catches of *G. swynnertoni* with traditional tsetse baits [17], [19]. Here, baiting devices with POCA did not affect their performance relative to one another; altogether results were remarkably constant between seasons at the same location and between the Serengeti and Mara. Considering the efficacy of the leg panels and targets, one should consider how much effort to invest in deploying and maintaining chemical baits (some of which are toxic, e.g. phenols) when it may be possible to adequately compensate by simply deploying more targets. In particular, the deployment of many small leg panels or targets with long-lasting insecticide impregnation may prove to be a cost-effective strategy. This approach would be particularly appropriate in conservation areas in East Africa, especially if fabrics could be engineered to be biodegradable after their effective lifespan.

### Pyramidal trap and 0.5 m^2^ target efficiency

As expected from many other studies on savannah tsetse, the pyramidal trap was found to be inherently inefficient as a trapping device for *G. swynnertoni*, i.e. less than two thirds of the flies that landed on attractive surfaces of the trap ended up being captured in the cage of the trap. This trapping rate is very similar in magnitude (31% efficiency) to that already measured for the S2 trap [17] where most *G. swynnertoni* that landed on the panels of the trap were never captured. In contrast to this, the pyramidal trap proved more efficient for *G. pallidipes*. There has been an underlying deficiency with traps for savannah species of tsetse. An absolute estimate of trapping efficiency is, however, difficult as there are many untested assumptions concerning fly behaviour and counting accuracy near traps that could affect the outcome. In contrast to this, the low number of flies (<2%) landing on transparent 0.5 m^2^ sticky targets compared to the number alighting on adjoining blue-black-blue cloth targets of the same size and shape suggests that very few *G. swynnertoni* or *G. pallidipes* circle the oblong 0.5 m^2^ targets before landing. Ndegwa and Mihok [17] used electric nets placed radially from the S2 trap and found that most (89%) *G. swynnertoni* flying in the vicinity of the trap circled within 0–1 m of it, and by covering the blue trap sides with adhesive fly rolls they found that 84% of approaching flies landed on the trap before entering it.

### Concluding remarks

Area-wide tsetse population suppression typically requires the deployment of many thousands of devices; devices need to be effective and inexpensive, and ideally should also be maintenance-free [40]. *G. swynnertoni* is abundant in areas with difficult logistics; it also presents limited options for dealing with as it occurs in protected areas frequented by large numbers of tourists. Simple targets that attract flies, which then land on insecticide-impregnated surfaces, are most suitable in this context. The most practical device for area-wide suppression of *G. swynnertoni* populations would be a large blue-black, insecticide-impregnated 1 m^2^ target. Our results show that there is no significant difference between the blue-black and blue-black-blue 1 m^2^ targets. A number of smaller targets in blue and black or all-blue leg panels with the same surface area would achieve the same result. Although all-blue leg panels would also provide a satisfactory control device a black element in the target is recommended where *G. swynnertoni* is the target species. The most cost-effective size of these devices and the associated costs of fabricating, deploying and maintaining large targets versus a higher number of small targets or leg panels still need to be determined in field trials. Our findings indicate that targets smaller than 0.5 m^2^ are not recommended for either *G. swynnertoni* or *G pallidipes*. Long-lasting but ultimately biodegradable devices of simple construction could be used to reduce disease transmission in the high-profile wildlife conservation areas of Tanzania and Kenya where *G. swynnertoni* is the main vector of human and animal trypanosomiasis. Either phthalogen or turquoise blue would be suitable for these visual control devices. Effective control will also require adaptive management [41] whereby tsetse populations are monitored and disease-transmission hot spots are identified for additional intervention. For long-term eradication goals, the detection of very low-density, residual pockets of tsetse is also critical [42]. The best monitoring tool would clearly be a leg panel or cloth target of equivalent size covered with adhesive film. Since this approach is not very economical or practical outside of a research context, all-blue plastic leg panels covered with insect glue can be used as an effective alternative.
